# Remote, Automated, and MRI-Compatible Administration of Interoceptive Inspiratory Resistive Loading

**DOI:** 10.3389/fnhum.2020.00161

**Published:** 2020-05-12

**Authors:** Sebastian W. Rieger, Klaas Enno Stephan, Olivia K. Harrison

**Affiliations:** ^1^Oxford Centre for Human Brain Activity, Wellcome Centre for Integrative Neuroimaging, Department of Psychiatry, University of Oxford, Oxford, United Kingdom; ^2^FMRIB Centre, Wellcome Centre for Integrative Neuroimaging, Nuffield Department of Clinical Neurosciences, University of Oxford, Oxford, United Kingdom; ^3^Translational Neuromodeling Unit, Institute for Biomedical Engineering, University of Zurich and ETH Zürich, Zurich, Switzerland; ^4^Wellcome Centre for Human Neuroimaging, University College London, London, United Kingdom; ^5^Max Planck Institute for Metabolism Research, Cologne, Germany; ^6^Nuffield Department of Clinical Neurosciences, University of Oxford, Oxford, United Kingdom

**Keywords:** interoception, breathing, perception, inspiratory resistance, MRI

## Abstract

Research on how humans perceive sensory inputs from their bodies (“interoception”) has been rapidly gaining momentum, with interest across a host of disciplines from physiology through to psychiatry. However, studying interoceptive processes is not without significant challenges, and many methods utilized to access internal states have been largely devoted to capturing and relating naturally occurring variations in interoceptive signals (such as heartbeats) to measures of how the brain processes these signals. An alternative procedure involves the controlled perturbation of specific interoceptive axes. This is challenging because it requires non-invasive interventions that can be repeated many times within a subject and that are potent but safe. Here we present an effective methodology for instigating these perturbations within the breathing domain. We describe a custom-built circuitry that is capable of delivering inspiratory resistive loads automatically and precisely. Importantly, our approach is compatible with magnetic resonance imaging (MRI) environments, allowing for the administration of complicated experimental designs in neuroimaging as increasingly required within developing fields such as computational psychiatry/psychosomatics. We describe the experimental setup for both the control and monitoring of the inspiratory resistive loads, and demonstrate its possible utilities within different study designs. This methodology represents an important step forward from the previously utilized, manually controlled resistive loading setups, which present significant experimental burdens with prolonged and/or complicated sequences of breathing stimuli.

## Introduction

A fundamental aspect of complex beings is the sensation, perception, and control of the physical body. “Interoception” can be considered as the processing of sensory inputs from the body by the nervous system across both subconscious and conscious domains (Khalsa et al., 2017). However, surprisingly little is known about the dynamic interplay between the brain and body beyond homeostatic reflex control (Pezzulo et al., 2015; Stephan et al., 2016; Petzschner et al., 2017), and the burgeoning field of interoceptive research has its sights set on understanding brain–body axes for potential applicability to emotions, decision making, consciousness, and mental health (recently reviewed by Khalsa et al., 2017), to name a few. In this article, we propose a significant methodological advancement regarding the experimental requirements for investigating interoception in the respiratory domain.

In order to study the brain–body interface across interoception, we need to equip ourselves with tools for capturing (and potentially inducing) perturbations in interoceptive experiences. This may take the form of measuring and modeling natural fluctuations that occur within and across interoceptive domains, such as variations in heart rate (Pollatos et al., 2007; Pappens et al., 2014), breath-to-breath variability in respiration (Paulus, 2013; Vlemincx et al., 2013), or irregularities across the gastric cycle (Herbert et al., 2012). Alternatively, we can consider actively invoking changes in these systems in a controlled, timely and reversible manner. Such active disturbances are critical for testing hypotheses about the principles that govern interoception. For example, current theories postulate that interoception obeys the same hierarchical Bayesian principles as exteroception (Seth, 2013; Pezzulo et al., 2015; Stephan et al., 2016; Petzschner et al., 2017). A critical prediction of these theories concerns the occurrence of specific prediction error signals (interoceptive surprise), e.g., reflected by activity of the insula, in response to unexpected changes in sensory inputs from the body. Testing this prediction requires experimental perturbations that elicit controlled prediction errors in a trial-by-trial fashion. Active perturbations of bodily states are also important for clinical applications (Stewart et al., 2014; Berner et al., 2017), not least in computational psychiatry/psychosomatics where model-based assessment of interoceptive surprise in individual patients plays a central role in proposals for differential diagnostics (Stephan et al., 2016; Petzschner et al., 2017).

Methods to induce controlled perturbations of interoception have so far been trialed in the cardiac domain (via intra-venous administration of isoproterenol Hassanpour et al., 2016), with breathing (via inspiratory and/or expiratory resistance; Paulus et al., 2012; Hayen et al., 2015, 2017; Berner et al., 2017; Faull and Pattinson, 2017), with gastric and bladder disturbances (via inflation of inserted balloons; Wang et al., 2008), or via baroreceptor manipulation (Owens et al., 2018). While pharmacologically-induced cardiac alterations provide strong effects and appeal to the wide body of research on natural fluctuations in interoceptive heart-related signals (Critchley et al., 2013; Garfinkel et al., 2015, 2016a,b; Quadt et al., 2018; Petzschner et al., 2019), the time-related decays required for returning to baseline as well as the burden and risks associated with repeated pharmacological interventions within the same subject pose restrictions on widespread application of this method. Similarly, the use of both gastric/bladder balloons and baroreceptor manipulations face challenges with regard to patient comfort and acceptance.

Here, we pursue a different approach for perturbing interoception in a fashion that is controlled, safe, repeatable, and not too inconvenient for human participants. We focus on respiration, presenting an advanced circuitry for the automated administration of inspiratory resistances. This circuitry builds on a previously published magnetic resonance imaging (MRI)-compatible inspiratory resistance circuit (Faull et al., 2016, 2017; Faull and Pattinson, 2017), incorporating computer-controlled solenoid valves for timely commencement and elimination of resistance, and an electronically controlled, flow-mediated inspiratory valve device (POWERbreathe, IMT Technologies Ltd., Birmingham, United Kingdom) to allow for adjustable maximal resistances. While the previous methodology has been employed to measure the brain activity [via functional MRI (fMRI)] related to the conditioned anticipation and perception of inspiratory resistive loads, these advances significantly aid the development of complicated protocols required for a more detailed study of dynamic brain–body interactions (Paulus et al., 2019). These protocol requirements often call for accurately controlled, repeatable, and changeable administration of interoceptive perturbations, which are essential to amass the necessary statistical evidence for quantifying subject-specific processes involved in interoception such as dynamic predictions and learning (Rescorla et al., 1972).

## Materials and Equipment

### Previous Inspiratory Resistance Administration Circuitry

**FIGURE 1:**
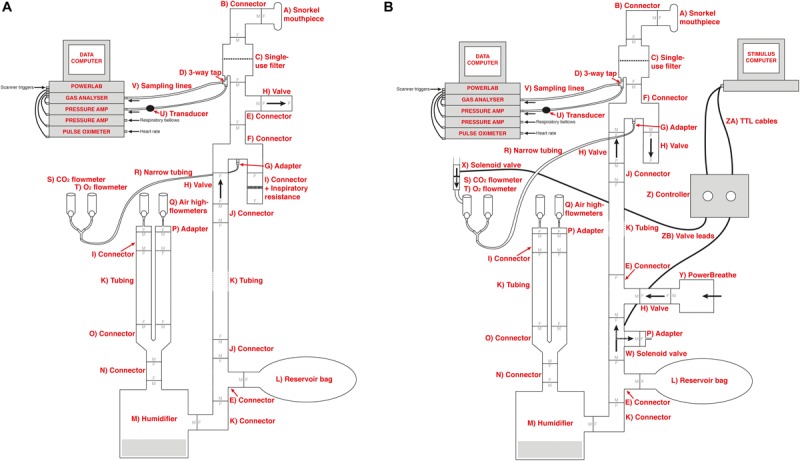
Schematic of the previously utilized inspiratory resistance circuit (Faull et al., 2016, 2017; Faull and Pattinson, 2017) (presented in **A**) and the new circuit design **(B)** that allows remote administrations of inspiratory resistance. In both systems, medical air is supplied to the subject, with a reservoir of 2 L. Excess flow and expiration escapes through a one-way valve (labeled H), close to the mouth to minimize rebreathing. A diving mouthpiece (labeled A) is connected to a bacterial and viral filter (labeled C), and sampling lines connect to a pressure transducer (labeled U) and amplifier (Pressure transducer indicator, PK Morgan Ltd., Kent, United Kingdom) for inspiratory pressure readings, and to a gas analyzer (via sampling line labeled V) (Gas Analyzer; ADInstruments Ltd., Oxford, United Kingdom) for respiratory gases. In **(A)**, resistive inspiratory loading is induced by discontinuing the delivery of medical air (via the flowmeter and emptying of the reservoir bag), forcing the subject to draw air through the resistor (porous glass disc labeled I). In **(B)**, resistive inspiratory loading is automatically achieved via the stimulus computer, whereby signals are sent through the parallel port to control valve 1 (labeled W) to redirect the supply of medical air to vent to the environment, forcing the subject to draw air through the POWERbreathe device (labeled Y). Periodically throughout scanning, small boluses of additional carbon dioxide (CO_2_) can be administered through manual control of the CO_2_ flowmeter (labeled S) in **(A)**, or automatic control via valve 2 (labeled X) in **(B)**, to raise the partial pressure of end-tidal CO_2_ (P_ET_CO_2_) to match the P_ET_CO_2_ rise induced by inspiratory loading periods. A final flowmeter (labeled T) is available for manual input of additional oxygen (O_2_) to the system. A full list of the labeled component parts can be found in the Supplementary Material.

The previous circuitry that has been employed to manually administer inspiratory resistances within an MRI scanner (Faull et al., 2016, 2017; Faull and Pattinson, 2017) is outlined in Figure 1A. In this experimental setup, compressed and humidified medical air was delivered to the participant via a breathing system, whereby air flow was maintained at a rate to adequately allow free breathing and access to an available air reservoir (within a reservoir bag) of 2 L. When a (conditioned) visual cue appeared on the screen, the delivery of compressed air was manually halted via closure of the corresponding air flowmeters, allowing the reservoir bag to empty over the course of approximately 3–8 s (anticipation period), followed by the application of inspiratory resistance once the air reservoir was empty. Additionally, delivery of supplementary gas mixtures [oxygen (O_2_) and a carbon dioxide (CO_2_) mix of 10% CO_2_, 21% O_2_, balance nitrogen] was supplied to the participant, allowing for periods of manual gas mixture administration (during rest periods) to decorrelate changes in expired CO_2_ and O_2_ from periods of inspiratory resistance. For participant safety when using the system, a new anti-bacterial, anti-viral, single-use filter is used for each participant. As this single-use filter is located within the pathways of both inspiration and expiration through the circuit, the filter protects both the participant from the system and the system from the participant. Therefore, after each use the filter is disposed of and everything between the filter and participant (the scuba mouthpiece and one elbow connector) is disinfected.

### Solenoid Valves

Within the new inspiratory resistance circuitry design (Figure 1B), two solenoid valves have been placed to allow for the automated and immediate application of inspiratory resistances, and administer small boluses of CO_2_ in a predetermined manner. Valve 1 (W in Figure 1B) is a wide-bore, direct-acting, 3/2-way solenoid valve (3/918-24/1002/R370-GN N.O. 24V DC, Buschjost Magnetventile GmbH & Co. KG, Germany) fitted with brass stem adaptors (MM052206N, John Guest Ltd.) to match the diameter of the air supply tubing, reducing the influence of any added baseline resistance to breathing. This valve is of the “normally open” type and will in its default state allow air to flow from the supply toward the mouthpiece. When actuated, the valve shuts off the port which supplies the mouthpiece and so initiates inspiratory loading. At the same time, the valve vents the medical air supply to the atmosphere to prevent overpressure. Valve 2 (X in Figure 1B) is a normally closed direct-acting 2/2-way valve (type 6013, Bürkert GmbH & Co. KG, Germany) that is inserted into the gas supply line for the CO_2_. This valve is closed by default, and when activated will open to allow the administration of the gas mixture for any adjustable length of time.

### Solenoid Valve Control Box

**FIGURE 2:**
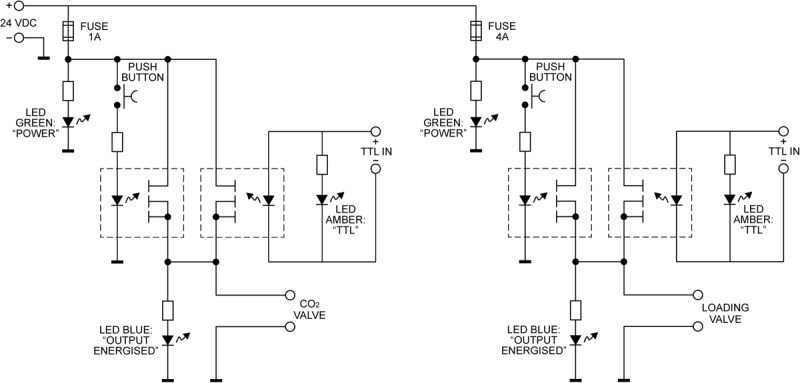
The valve controller circuit. Each of the two solenoid valves is switched using a pair of Panasonic AQZ102 optoFET relays connected in parallel, allowing them to be energized individually, either manually via the pushbutton, or from a TTL signal, while ensuring the TTL input remains galvanically separated from the control circuit. Power is supplied from a standard 24VDC power supply (Meanwell GST90A24-P1M, not shown). For additional protection from transients at the outputs, the valves are connected using cable assemblies with built in suppression (Murrelektronik 7000-40881-6360200, not shown).

The solenoid valves are operated via a custom-built control circuitry box (Figure 2), which can be interfaced with a presentation software program such as PsychToolBox (Borgo et al., 2012), or activated manually via a button press on the control box. The valve controller uses a 24 V DC 5 A power supply switched using optically coupled solid state (photoMOS) relays (AQZ102, Panasonic). Each output is connected to the supply via two parallel relays, one of which can be operated via a pushbutton, while the other one is energized directly from the TTL compatible control input. This allows standalone manual operation (without a PC connected) as well as manual override (i.e., manual operation with a PC connected), while ensuring the TTL inputs remain galvanically separated from the power supply and valve circuits. Kickback protection is achieved by the use of suitable solenoid valve leads (40881 Series, Murrelektronik).

To interface with PsychToolBox (or other presentation programs), simple commands can be sent via the parallel port of the presentation computer and be fed directly into the control box. This feature allows for completely automated resistance administration, which could either be using a predetermined, randomized, or online-updating sequence of stimuli. Sample code for control via the parallel port is provided in the Supplementary Material.

### Adjustable Maximal Resistance

To allow for an adjustable maximal inspiratory resistance, the porous glass disc previously used to evoke an inspiratory resistance (Figure 1A) has been replaced with an electronically controlled, flow-mediated resistance valve (POWERbreathe, IMT Technologies Ltd., Birmingham, United Kingdom). This device can be set to a maximal inspiratory pressure value between 3 and 200 cmH_2_O using a variable aperture to create a static resistance to inspiratory flow. When the pressure generated by the participant inspiring against the POWERbreathe valve exceeds this set maximal pressure value, the valve is released. In addition, pressure at the mouth is measured within the breathing system, allowing a direct recording of the inspiratory pressure generated voluntarily against the external resistance by the participant. However, if a measure of inspiratory *resistance* would be required, and additional spirometer flowhead (and associated differential transducer; ADInstruments Ltd., Oxford, United Kingdom) could be easily inserted into the system between the filter (labeled C in Figure 1) and connector leading to inspiratory and expiratory valves (labeled F in Figure 1).

## Methods

### Example Participant

One example participant gave written, informed consent to demonstrate the use of this equipment. Ethical approval granted by the Zurich Ethics Committee.

### Resistance Administration

This circuit design provides a platform from which a number of inspiratory resistance protocols are compatible and possible. One of the simpler methodologies would be to employ an on/off binary protocol, where the maximal resistance on the POWERbreathe is set to a percentage of each participant’s maximum inspiratory pressure (which can be tested using a standardized maximal inspiratory pressure protocol on the POWERbreathe device). To demonstrate this, we conducted physiological recordings during repeated applications of an inspiratory resistance value of 55 cmH_2_O, which was determined by calculating 70% of the participant’s maximal inspiratory pressure of 79 cmH_2_O. The participant was instructed to maintain normal breathing depth and rate during the resistive loading periods.

Alternatively, graded levels of inspiratory resistance could be employed via a pre-determined protocol input to the POWERbreathe, although visual feedback may be a necessity here to ensure participants reach the desired inspiratory pressure in each trial. To demonstrate this protocol, we repeatedly activated valve 1 with 5 cmH_2_O graded increases in set pressure from 5 to 30 cmH_2_O.

### Administration of Additional Gas Mixtures

To demonstrate the addition of specific gas mixtures into the system, we conducted physiological recordings during the administration of small boluses of elevated CO_2_. To achieve these boluses, valve 2 was repeatedly activated for a short (∼1 s) duration, whereby a gas mixture containing 20% CO_2_, 21% O_2_ and the balance nitrogen was released into the system with a maximal flow-rate of 2 L/min (controlled by the flowmeter labeled “S” in Figure 1B). Any approved gas mixture could be added into the system for any specified duration, and example code to control each of the valves automatically is provided in the Supplementary Material.

## Results

### Resistance Administration

**FIGURE 3:**
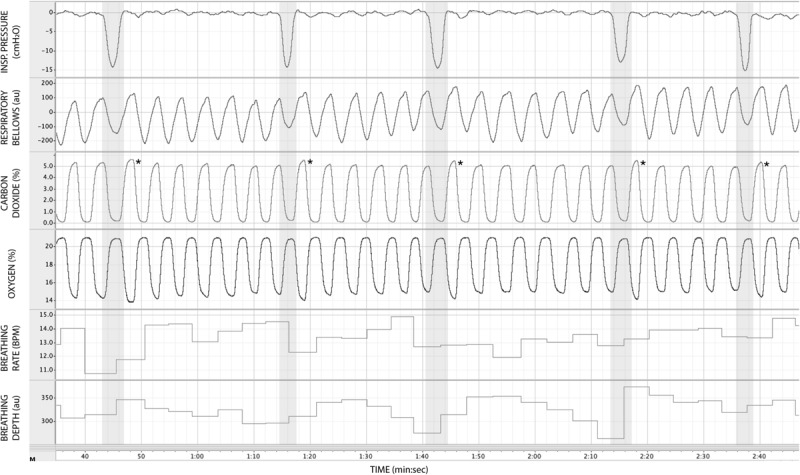
Physiological traces recorded during the application of five inspiratory resistance periods (gray areas), where the maximal inspiratory resistance was set at 55 cmH_2_O. Asterisks (*) denote an increase in expired pressure of end-tidal carbon dioxide (P_ET_CO_2_—determined by the peak values at the end of each expiration in the CO_2_ trace) immediately following each inspiratory resistance period.

**FIGURE 4:**
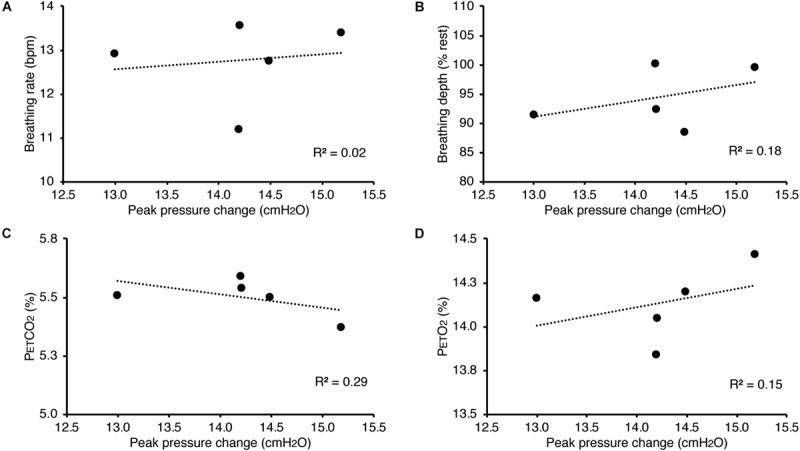
Relationship between the inspiratory pressures generated (from Figure 3) against a consistent inspiratory resistive load and the additional physiological variables measured. No significant relationships were found between the fluctuations in inspiratory pressure and breathing rate **(A)**, breathing depth (measured as a change from resting values, **B**), pressure of end-tidal carbon dioxide (P_ET_CO_2_, **C**), and pressure of end-tidal oxygen (P_ET_O_2_, **D**).

In Figure 3, we demonstrate example physiological traces recorded during the repeated activation of valve 1, and subsequent inspiratory resistance applications for a set value (here 55 cmH_2_O). This protocol allows a very clear perceptual distinction between the presence and absence of an inspiratory resistance, and could then be programmed and automated to fulfill the requirements of the research question at hand. Furthermore, as the pressure generated on each trial results from the inspiratory effort produced by the participant, either visual biofeedback could be employed to ensure pressure consistency in each trial, or alternatively the natural variability in both inspiratory pressure and perceptual ratings across trials could be used to tease apart differences in physiological vs. perceptual brain activity (for example). This example additionally demonstrates the perturbations in expired end-tidal carbon dioxide (P_ET_CO_2_) that immediately follow each resistance stimulus. The relationship between the inspiratory pressure generated in each stimulus and the remaining physiological measures is demonstrated in Figure 4, whereby no significant associations were observed between inspiratory pressure and breathing rate, depth, P_ET_CO_2_ nor P_ET_O_2_ for this example participant. The differences between the average changes in physiological variables during constant inspiratory resistive loading and baseline rest periods are presented in Table 1, whereby inspiratory resistance induced significant increases in P_ET_CO_2_ and decreases in P_ET_O_2_, with marginal decreases in both breathing rate and depth in this example participant.

**TABLE 1:** Effect of constant levels of inspiratory loading on the physiological variables measured.

	**Inspiratory pressure (cmH_2_O)**	**Breathing rate (bpm)**	**Breathing depth (au)**	**Pressure of end-tidal CO_2_ (%)**	**Pressure of end-tidal O_2_ (%)**
*Mean values:*					
Rest periods	0.92 (0.20)	13.34 (0.79)	327.00 (12.16)	5.18 (0.12)	14.71 (0.21)
Inspiratory loading	14.21 (0.79)	12.77 (0.94)	308.80 (16.91)	5.50 (0.08)	14.13 (0.21)
Difference *P*-value	<0.001	0.06	0.05	<0.001	<0.001
*Relationship with inspiratory pressure:*					
Regression *R*^2^	−	0.02	0.18	0.29	0.15
Coefficient	−	0.12	0.06	−5.25	1.49
Degrees of freedom (total)	−	4	4	4	4
*P*-value	−	0.81	0.48	0.34	0.51

*Mean values (±SD) for resting periods (between stimuli) and inspiratory loading periods are presented in the top panel, and regression results for each variable (Y) against inspiratory pressure (X) in the bottom panel (included values were averaged across each inspiratory loading period).*

**FIGURE 5:**
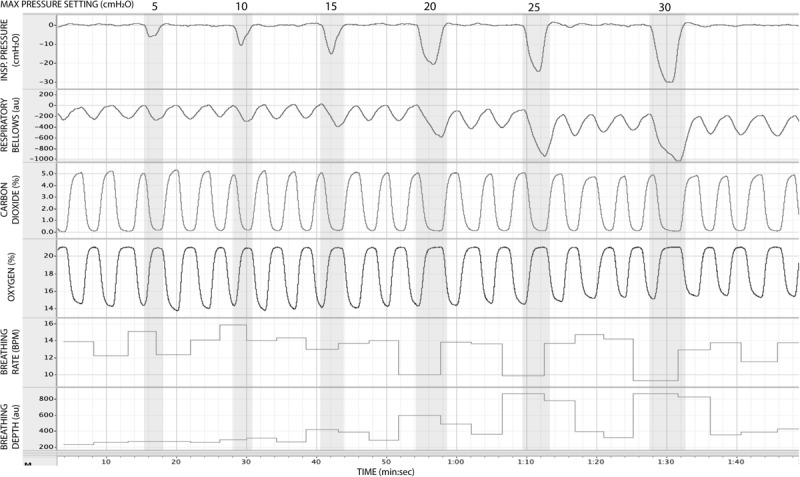
Physiological traces recorded during the application of five inspiratory resistance periods (gray areas), where the level of inspiratory resistance was graded at intervals of 5 cmH_2_O between 5 and 30 cmH_2_O.

**FIGURE 6:**
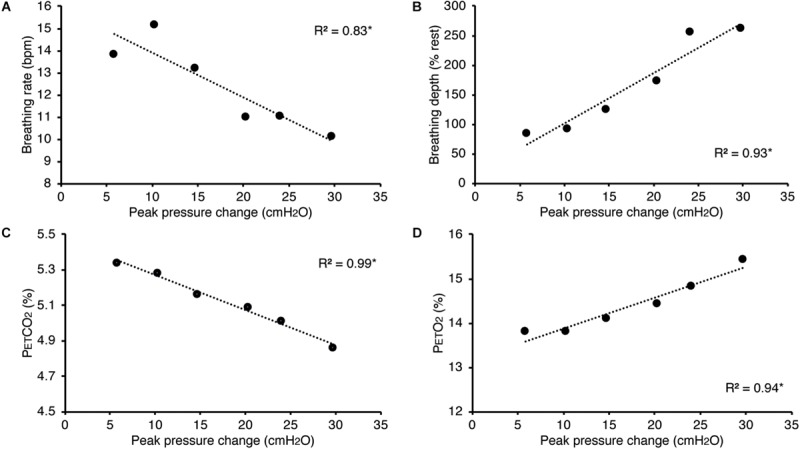
Relationship between the inspiratory pressures generated (from Figure 5) against a graded inspiratory resistive load and the additional physiological variables measured. Significant relationships (denoted by *) were found between graded inspiratory pressure and breathing rate **(A)**, breathing depth (measured as a change from resting values, **B**), pressure of end-tidal carbon dioxide (P_ET_CO_2_, **C**), and pressure of end-tidal oxygen (P_ET_O_2_, **D**).

In Figure 5, we present example results from a graded increase in inspiratory resistance across stimuli. Overcoming these resistances requires a matched graded increase in inspiratory muscular effort, and for greater resistances, this may be achieved by employing deeper and/or longer breaths. This strategy is exemplified in Figure 5, whereby the distention on the respiratory bellows increases with larger resistances, demonstrating an increase in the inspiratory depth required to overcome the set resistance and a matching compensatory decrease in breathing rate. The relationship between the increase in inspiratory pressure generated against these resistances and the remaining physiological measures is demonstrated in Figure 6 and Table 2, whereby significant associations were observed between inspiratory pressure and breathing rate, depth, P_ET_CO_2_, and P_ET_O_2_ in this example participant.

**TABLE 2:** Regression relationships between the inspiratory pressures generated (from Figures 5, 6) when inspiring against a graded inspiratory resistive load (X) and the additional physiological variables (Y) measured (included values were averaged across each inspiratory loading period).

	**Breathing rate (bpm)**	**Breathing depth (au)**	**Pressure of end-tidal CO_2_ (%)**	**Pressure of end-tidal O_2_ (%)**
Regression *R*^2^	0.83	0.93	0.99	0.94
Coefficient	−4.12	0.11	−50.11	13.51
Degrees of freedom (total)	5	5	5	5
P value	0.01	<0.01	<0.001	<0.01

### Administration of Additional Gas Mixtures

**FIGURE 7:**
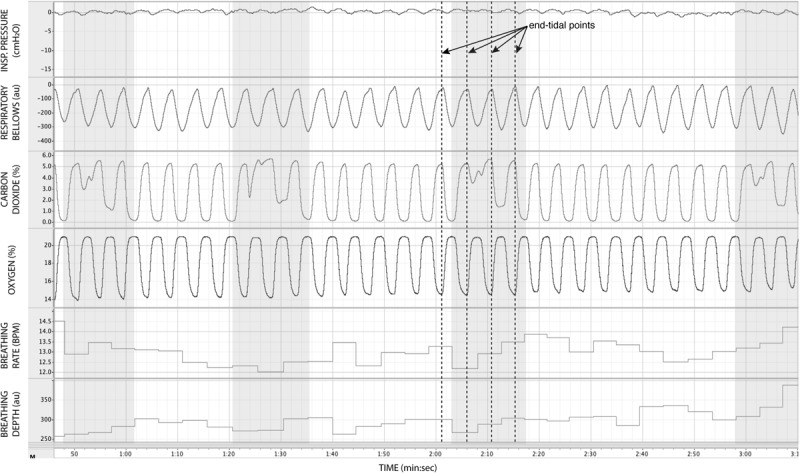
Physiological traces recorded during the application of four boluses of a carbon dioxide mixture (gray areas: 20% CO_2_, 21% O_2_, and the balance nitrogen) added to the medical air supply. Dotted lines represent example end-expiratory points, from which end-tidal CO_2_ and O_2_ values would be taken.

In Figure 7, we demonstrate the physiological traces recorded during repeated activation of valve 2, here allowing the administration of a gas mixture containing elevated levels of CO_2_. Importantly, the end-tidal values of expired CO_2_ (the peak value at the end of expiration, highlighted in Figure 7) can be seen to rise and then recede over the course of 2–3 breaths, which could be extended by specifying a longer duration of administration of the gas mixture. It is also observed that the CO_2_ value between breaths does not return to baseline, as the partial pressure of the CO_2_ within the gas mixture has been temporarily increased. Minimal effects from this administration are observed in expired O_2_, as well as breathing rate and depth (results presented in Table 3).

**TABLE 3:** Effect of adding small boluses of carbon dioxide (CO_2_) into the breathing circuit on the physiological variables measured.

	**Inspiratory pressure (cmH_2_O)**	**Breathing rate (bpm)**	**Breathing depth (au)**	**Pressure of end-tidal CO_2_ (%)**	**Pressure of end-tidal O_2_ (%)**
*Mean values:*					
Rest periods	1.07 (0.37)	12.85 (0.15)	284.37 (30.34)	5.26 (0.05)	14.52 (0.26)
CO_2_ periods	0.71 (0.24)	12.99 (0.56)	295.72 (31.78)	5.61 (0.10)	14.49 (0.44)
Difference *P*-value	0.16	0.66	0.62	<0.001	0.91
*Relationship with end-tidal CO_2_:*					
Regression *R*^2^	0.40	0.03	<0.01	.	0.10
Coefficient	1.06	−0.31	0.82	−	−0.57
Degrees of freedom (total)	7	7	7	−	7
*P*-value	0.09	0.70	0.99	−	0.43

*Mean values (±SD) for resting periods (between stimuli) and inspiratory loading periods are presented in the top panel, and regression results for each variable (Y) against end-tidal CO_2_ (X) in the bottom panel (included values were averaged across each rest and CO_2_ stimulus period).*

## Discussion

In this report, we have presented a custom-designed circuitry to administer automated, remote, timely, and repeated inspiratory resistances suitable for an MR environment. In this design, we have included the use of solenoid valves to control the administration of both inspiratory resistance and boluses of additional gas mixtures, which can be triggered either from a stimulus computer or by a manual button press on a custom-built circuitry box. This design represents a significant improvement from previously published methodologies (Faull et al., 2016, 2017; Faull and Pattinson, 2017), whereby inspiratory resistances were administered via manual closing of flowmeters and emptying of reservoir bags, both of which present substantial obstacles as the necessity of more complicated experimental designs beckons. As outlined in Section “Introduction,” methodologies such as the one described in this paper represent an essential step in our progression toward understanding the links between brain and body, and the importance of interoceptive processes across a wide range of neuroscientific domains.

The circuit design presented here has been specifically tailored to cater for the demands of an MRI environment. In this setting, where all equipment within the scanner room is required to be non-magnetic, the solenoid valves, electrical circuitry, gas flow control, and inspiratory resistance device can all be kept within the scanner control room, with flexible lengths of tubing allowing for the remote delivery of air supply and gas mixtures to scanners of variable distances from a control room port. All equipment within the scanner room is made of plastic or non-ferromagnetic materials, and both participants and the circuit are protected via a disposable, single-use bacterial and viral filter. Additionally, the sampling of gas and inspiratory pressures is fed back via sampling lines to monitoring devices that are also housed within the control room. To account for any gas sampling time-delays caused by the length of sampling line required, delays can simply be measured and the temporal shift incorporated into the analysis of any recorded physiological traces.

The advances made in this circuit design allow for implementation of a wide number of experimental procedures and inspiratory resistance protocols. While simple, binary protocols (such as the presence or absence of a noticeable inspiratory resistance) are one option, graded resistances are also possible with the use of the POWERbreathe device. Notably, the resistance to breathing set by the POWERbreathe does not necessarily determine the inspiratory pressure generated by the participant (recorded at the mouth), but rather a maximal pressure against which they inspire. As only initial ideas, we would postulate that this experimental setup could be utilized in one of three ways, namely: (1) using graded inspiratory resistances (within a limited range), where the participant is instructed to inspire using enough force to overcome the maximal pressure set on each trial (ensuring the external POWERbreathe inspiratory pressures are reached), (2) using a static (low or moderate) inspiratory pressure at the POWERbreathe, with participant instructions in the same manner as option 1 to ensure consistent pressures are generated at each trial, or (3) using a static, insurmountable pressure setting on the POWERbreathe, and simply measuring the natural variability that is inherent in the (voluntarily generated) inspiratory pressure readings (as demonstrated in Figure 3).

Different experimental options would need to be carefully considered in light of the specific study aim and also the potential confounding variables that each may entail. As can be seen from the example physiological traces provided in Figure 6, employing graded resistances almost perfectly induces corresponding changes in breathing rate and depth (to overcome the resistance), and expired P_ET_CO_2_ and P_ET_O_2_ in this instance. However, the relationship between these variables will be specific to the protocol used and the voluntary breathing actions taken by the participants. Therefore, the statistical analyses presented here are intended as a guide for how these could be assessed in different experimental scenarios, rather than a reference. While the system has methods for removing brain signal associated with pressure of expired gases (discussed below), the changes in breathing rate and depth may induce correlated head motion and associated image artifacts (if used in conjunction with brain imaging measures). Although established methods exist to remove physiological noise from brain imaging data (Brooks et al., 2013) (such as physiological noise modeling (PNM) in FSL; Brooks et al., 2008, and PhysIO for SPM; Kasper et al., 2017), this regression may partially remove neural signals of interest. While option 2 would allow a more constant change in corresponding physiology across trials, the latter option 3 has the potential advantage of decorrelating physiological changes from the perception of resistance to inspiration. As the interoceptive signal induced by this design will be composed of both the strength of the external resistance (i.e., how difficult it feels to inhale), as well as the motor recruitment response employed to overcome or inspire against that resistance, the dynamic nature of the latter may let us decorrelate the physiological artifacts from the static perception of the inspiratory resistance. In this scenario, regressing out the physiological artifacts (such as breathing rate and depth, head motion, etc.) would still leave the brain signal associated with the difficulty of inspiring against the resistance. Furthermore, the variability within the physiological signals could be compared to additional measures such as subjective perceptual ratings of work, effort, and/or anxiety across trials, allowing us to better understand which aspects of the voluntary motoric response may be associated with different aspects of interoceptive perception. However, one final note is that, due to the voluntary input into breathing circuitry, it is also possible that participants may choose to hold their breath and miss the interoceptive stimulus entirely. This scenario can be detected by both the inspiratory pressure and breathing belt traces, and we highly recommend collecting a rating of breathing difficulty (or similar) following each stimulus for confirmation of this event. These stimuli could then be accounted for in any analysis of behavioral and/or brain imaging data.

The design of this circuit also provides a means to tackle one of the key issues when utilizing breathing-related tasks in an fMRI protocol: Namely, the disturbances in blood gas concentrations and their subsequent effects on the blood oxygen level-dependent (BOLD) signal that is the fundament of most functional imaging sequences. The partial pressure of CO_2_ (PCO_2_) in the blood is a potent vasodilator, and tasks that induce changes in blood gases via altering natural breathing rhythms can thus either wash-out BOLD signal (via increases in PCO_2_) or induce global gray-matter vasoconstriction (via decreases in PCO_2_) that may interact non-linearly with the neurally induced BOLD response (Chang and Glover, 2009). Additionally, changes in the partial pressure of oxygen (PO_2_) in the blood also play a vasodilatory role; however, the magnitude of this response is considerably smaller than that induced by PCO_2_ (Chang and Glover, 2009). In this circuit design, we are first able to remotely measure the changes in expired CO_2_ and O_2_, whereby the end-tidal values are assumed to be indicative of alveolar gas (which closely parallels arterial carbon dioxide levels under normal ventilation-perfusion matching; Phan et al., 1987). Second, solenoid-valve controlled boluses of additional CO_2_ can also be delivered whenever required, for example, in some rest periods of the protocol, which allows the changes in expired gas to be decorrelated from the breathing task (Faull et al., 2015). However, it should be noted that maintenance of stable end-tidal gas measurements via end-tidal forcing (Wise et al., 2007) has not currently been incorporated into this system, although this could be integrated in place of the binary solenoid CO_2_ valve if necessary.

## Conclusion

Here we have presented a circuitry that has been designed to automatically administer periods of inspiratory resistance in a remote environment (such as an MRI scanner), allowing the potential instigation of more complex paradigms that probe the relationship between brain and body. However, while breathing represents a somewhat more easily accessible avenue in which to study interoception, the changes in blood gas pressures that often result from breathing tasks cannot be overlooked. Therefore, in this design, we have incorporated methods for both recording and decorrelating fluctuations in the pressure of expired CO_2_ and O_2_, as a representation of fluctuations in arterial gas pressures. Lastly, the use of an electronically controlled inspiratory resistance device allows for specification of a ceiling of maximal inspiratory resistances, although careful use of biofeedback may be necessary to ensure graded levels of pressure are reached by the participant on each trial if required. We hope that this circuit design may aid the more nuanced study of brain–body interactions and interoception in the respiratory domain.

## Data Availability Statement

The datasets generated for this study are available on request to the corresponding author.

## Ethics Statement

The studies involving human participants were reviewed and approved by Kanton Zürich Kantonale Ethikkommission. The patients/participants provided their written informed consent to participate in this study.

## Author Contributions

SR designed and built the automated valve system and contributed to the drafting and editing of the manuscript. KS oversaw the design and production of all methodological equipment and contributed to the drafting and editing of the manuscript. OH contributed to the design and production of all methodological equipment and contributed to the drafting and editing of the manuscript.

## Conflict of Interest

The authors declare that the research was conducted in the absence of any commercial or financial relationships that could be construed as a potential conflict of interest.
