# Supplementary material for: Remote, Automated, and MRI-Compatible Administration of Interoceptive Inspiratory Resistive Loading
Source: Front Hum Neurosci. 2020 May 12;14:161. doi: 10.3389/fnhum.2020.00161 (PMC7236550; doi:10.3389/fnhum.2020.00161)
Supplement: Supplementary file 1 [file Data_Sheet_1.docx]

Supplementary Material: Remote, automated and MRI-compatible administration of interoceptive inspiratory resistive loading

Sebastian W Rieger^1,2^, Klaas Enno Stephan^3,4,5^ and Olivia K. Harrison^3,6^

^1^ Oxford Centre for Human Brain Activity, Wellcome Centre for Integrative Neuroimaging, Department of Psychiatry, University of Oxford, Oxford, United Kingdom

^2^ FMRIB Centre, Wellcome Centre for Integrative Neuroimaging, Nuffield Department of Clinical Neurosciences, University of Oxford, Oxford, United Kingdom

^3^ Translational Neuromodeling Unit, Institute for Biomedical Engineering, University of Zurich and ETH Zürich, Zürich, Switzerland

^4^ Wellcome Centre for Human Neuroimaging, University College London, London, United Kingdom

^5^ Max Planck Institute for Metabolism Research, Cologne, Germany

^6^ Nuffield Department of Clinical Neurosciences, University of Oxford, Oxford, United Kingdom

# Equipment details

This manuscript and associated supplementary material provide the necessary information to source and assemble all major components of the breathing system, and the circuit details required for the controller box. Any further enquires can be directed to either Sebastian Rieger ([sebastian.rieger@psych.ox.ac.uk](mailto:sebastian.rieger@psych.ox.ac.uk)) or Olivia Harrison (née Faull, [faull@biomed.ee.ethz.ch](mailto:faull@biomed.ee.ethz.ch)).

Figure 1 (reproduced from manuscript for convenience). Schematic of the previously-utilised inspiratory resistance circuit^16,26^ (presented in panel A) and the new circuit design (panel B) that allows remote administrations of inspiratory resistance. In both systems, medical air is supplied to the subject, with a reservoir of 2 L. Excess flow and expiration escapes through a one-way valve (labelled H), close to the mouth to minimise rebreathing. A diving mouthpiece (labelled A) is connected to a bacterial and viral filter (labelled C), and sampling lines connect to a pressure transducer (labelled U) and amplifier (Pressure transducer indicator, PK Morgan Ltd, Kent, UK) for inspiratory pressure readings, and to a gas analyser (via sampling line labelled V) (Gas Analyser; ADInstruments Ltd, Oxford, United Kingdom) for respiratory gases. In diagram A, resistive inspiratory loading is induced by discontinuing the delivery of medical air (via the flowmeter and emptying of the reservoir bag), forcing the subject to draw air through the resistor (porous glass disc labelled I). In diagram B, resistive inspiratory loading is automatically achieved via the stimulus computer, whereby signals are sent through the parallel port to control valve 1 (labelled W) to redirect the supply of medical air to vent to the environment, forcing the subject to draw air through the POWERbreathe device (labelled Y). Periodically throughout scanning, small boluses of additional carbon dioxide (CO_2_) can be administered through manual control of the CO_2_ flowmeter (labelled S) in diagram A, or automatic control via valve 2 (labelled X) in diagram B, to raise the partial pressure of end-tidal CO_2_ (P_ET_CO_2_) to match the P_ET_CO_2_ rise induced by inspiratory loading periods. A final flowmeter (labelled T) is available for manual input of additional oxygen (O_2_) to the system. A full list of the labelled component parts can be found in the supplementary material.

**Supplementary Table 1**. Equipment details for Figure 1

| A | Scubapro super comfort regulator mouthpiece | https://www.mikesdivestore.com/products/scubapro-super-comfort-mouthpiece |
| --- | --- | --- |
| B | Intersurgical elbow 22F-22M | http://www.intersurgical.co.uk/products/critical-care/connectors-and-elbows |
| C | GVS Filter (8866/01BAUA) | http://www.gvs.com/product-family/191/800/8866 |
| D | 3-way tap | e.g. https://www.medical-world.co.uk/p/iv-accessories-dressings-furniture/intravenous-accessories/tap-3-way-anaesthetic-stopcock-with-facility-for-tube-disposable-sterile-single-use-x-1/3645 |
| E | Intersurgical T-piece 22M-22M-22F | http://www.intersurgical.co.uk/products/critical-care/connectors-and-elbows |
| F | Intersurgical Y-piece swivel connector 22M-22M-22M | http://www.intersurgical.co.uk/products/critical-care/connectors-and-elbows |
| G | Luer to hose barb adapter | e.g. https://www.coleparmer.co.uk/i/cole-parmer-male-luer-with-lock-ring-x-1-4-hose-barb-nylon-pack-of-25/4550519 |
| H | Hans Rudolf one-way valve | http://www.rudolphkc.com/img/uploads/pdf/691125%200714%20O.pdf |
| I | Intersurgical connector 22F-22F with inserted custom porous glass disc | e.g. https://www.heraeus.com/en/hca/products_hca/plates_quartz_1/  filter_discs_1/filter_discs_hca.html |
| J | Intersurgical connector 22M-22M | http://www.intersurgical.co.uk/products/critical-care/connectors-and-elbows |
| K | Intersurgical tubing 22F 22mm | http://www.intersurgical.co.uk/products/anaesthesia/scavenging-tube |
| L | Intersurgical reservoir bag 2L 2F neck | http://www.intersurgical.co.uk/products/anaesthesia/aagbi-test-reservoir-bag |
| M | Fisher & Paykel humidifier | https://www.fphcare.co.nz/mr810/ |
| N | Intersurgical connector 22F-22F | http://www.intersurgical.co.uk/products/critical-care/connectors-and-elbows |
| O | Intersurgical Y-piece connector 22M-22M-22M | http://www.intersurgical.co.uk/products/critical-care/connectors-and-elbows |
| P | Intersurgical connector 22F- oxygen stem | http://www.intersurgical.co.uk/products/critical-care/connectors-and-elbows |
| Q | Gas Arc flowmeter 40 L/min | https://www.hisltd.co.uk/ProductDetail/Gas-Arc-Flowmeter/c67d8962-4ae6-4a97-8c7c-a9956d035ec8 |
| R | Bunzl healthcare 5mm bubble tubing | https://catalogue.bunzlhealthcare.co.uk/product/suction-bubble-tubing-non-sterile/ |
| S | Therapy Equipment railmounted carbon dioxide flowmeter | http://www.therapyequipment.co.uk/product/Diamond-Flowmeter-Range/products_flowmeters/index.html |
| T | Therapy Equipment railmounted oxygen flowmeter | http://www.therapyequipment.co.uk/product/Diamond-Flowmeter-Range/products_flowmeters/index.html |
| U | ADInstruments pressure transducer | https://www.adinstruments.com/products/bp-transducercable-kit |
| V | Intersurgical gas monitoring line | https://www.intersurgical.com/products/critical-care/gas-monitoring-lines |
| W | Buschjost Magnetventile GmbH & Co large bore solenoid valve for inspiratory loading | Type: Normally open 3/918-24/1002/R370-GN N.O. 24V DC  https://www.buschjostventile.de/attachments/1990a721-3afe-4099-a5e4-148763561294/de/Gewindeventile_112017.pdf  Plus required fittings (3 pcs.): MM052206N, John Guest Ltd. |
| X | Bürkert GmbH & Co solenoid valve for CO_2_ | Type: Normally closed direct-acting 2/2-way valve, type 6013  https://www.burkert.co.nz/en/type/6013  Plus required fittings (one each): 1/8" BSP'P' (cone seat) swivel female x 1/4" BSP'T' male adaptor, Part number FST-2BT-02-04, Custom Fittings Ltd., UK; British standard gas distinct direct fitting probe for 4 bar air with male 1/8" BSPP (cone seat) thread (part number 8502-01), Therapy Equipment, UK; British standard gas distinct single Schrader valve for 4 bar air with male 1/4" BSPT thread (part number 6102/P), Therapy Equipment, UK |
| Y | PowerBreathe device | https://www.powerbreathe.com/index.php |
| Z | Custom built controller box | See Figure 2 for details |
| ZA | TTL signal cables | DB25-to-BNC adapter (made in-house) and two generic BNC leads of appropriate length (e.g. part number L00015A1455, Telegärtner, for 10 metre leads) |
| ZB | Valve leads | 40881 Series cable assembly, part number 7000-40881-6360200, Murrelektronik |

Note: Approximate cost of the entire system is ~£25,000 (depending on local suppliers)

# Example Matlab code for the control of the circuit box

This code is suitable for use with a computer that has a parallel port, and requires the University of South Dakota Internet Psychology Lab's MEX file plug-in (available from http://apps.usd.edu/coglab/psyc770/IO64.html). It is assumed that the TTL signals controlling the valves are taken from pins 2 and 3. If a computer without a parallel port is employed, a commercially available digital I/O-card may be used instead to produce the TTL signals, and the code adapted accordingly.

% Parallel port tester for valve control box

% Sebastian W Rieger

% Wellcome Centre for Integrative Neuroimaging (WIN)

% University of Oxford

% 02.05.2019

% Initialise port (if this fails, you need to install the mex-file plugin):

config_io;

% Set port address (you can find this in the Windows device manager):

port_address = hex2dec('378');

% Energise the valve connected to pin 2 on the parallel port:

outp(port_address, 1);

% Wait for an appropriate amount of time (3 seconds in this example):

pause(3);

% De-energise the valves:

outp(port_address, 0);

% Energise the valve connected to pin 3 on the parallel port:

outp(port_address, 1);

% Wait for an appropriate amount of time (3 seconds in this example):

pause(3);

% De-energise the valves:

outp(port_address, 0);
